# Whole Genome Pathway Analysis Identifies an Association of Cadmium Response Gene Loss with Copy Number Variation in Mutant p53 Bearing Uterine Endometrial Carcinomas

**DOI:** 10.1371/journal.pone.0159114

**Published:** 2016-07-08

**Authors:** Joe Ryan Delaney, Dwayne G Stupack

**Affiliations:** Department of Reproductive Medicine, Division of Gynecologic Oncology, Rebecca and John UCSD Moores Cancer Center, La Jolla, California, United States of America; University of Navarra, SPAIN

## Abstract

**Background:**

Massive chromosomal aberrations are a signature of advanced cancer, although the factors promoting the pervasive incidence of these copy number alterations (CNAs) are poorly understood. Gatekeeper mutations, such as p53, contribute to aneuploidy, yet p53 mutant tumors do not always display CNAs. Uterine Corpus Endometrial Carcinoma (UCEC) offers a unique system to begin to evaluate why some cancers acquire high CNAs while others evolve another route to oncogenesis, since about half of p53 mutant UCEC tumors have a relatively flat CNA landscape and half have 20–90% of their genome altered in copy number.

**Methods:**

We extracted copy number information from 68 UCEC genomes mutant in p53 by the GISTIC2 algorithm. GO term pathway analysis, via GOrilla, was used to identify suppressed pathways. Genes within these pathways were mapped for focal or wide distribution. Deletion hotspots were evaluated for temporal incidence.

**Results:**

Multiple pathways contributed to the development of pervasive CNAs, including developmental, metabolic, immunological, cell adhesion and cadmium response pathways. Surprisingly, cadmium response pathway genes are predicted as the earliest loss events within these tumors: in particular, the metallothionein genes involved in heavy metal sequestration. Loss of cadmium response genes were associated with copy number changes and poorer prognosis, contrasting with 'copy number flat' tumors which instead exhibited substantive mutation.

**Conclusion:**

Metallothioneins are lost early in the development of high CNA endometrial cancer, providing a potential mechanism and biological rationale for increased incidence of endometrial cancer with cadmium exposure. Developmental and metabolic pathways are altered later in tumor progression.

## Introduction

Cancer genomes can evolve through acquisition of favorable mutations, copy number variations, or both. Determination of those mutations which recur on individual genes or even codons of individual amino acids within a gene has aided the development of targeted therapeutics, such as Vemurafenib to target BRAF V600E mutant tumors. The efficacy of targeting of mutations is improved for those mutations which arise early in tumor development (“trunk” mutations) and thus occupy the vast majority of tumor cells [1]. However, not all tumors have a clear trunk mutation or they have trunk mutations which cannot currently be targeted. For example, the mutational signature of serous ovarian cancers clustered away from 11 other cancer types due to the lack of any tumor suppressor or oncogene mutation other than p53 [2].

Copy number alterations (CNAs) are another route to oncogenesis. Some dramatic alterations, such as massive amplification of *HER2* [3], have been clearly linked to tumor promoting roles. Yet again many tumors do not present such clearly oncogenic alterations of a single locus, but rather have much smaller alterations spread throughout their genome. Chromosome arms are allelically deleted or amplified based on their content of modest-effect tumor suppressors or oncogenes, respectively [4]. Mutation or loss of well-known tumor suppressors p53 [5, 6] or *BRCA1/2* [7] can contribute to the initiation of widespread aneuploidy and CNAs, however p53 or *BRCA* mutant tumors do not always display a largely altered copy number landscape. There must be other genetic changes which enable certain tumors to acquire a larger burden of CNAs.

One cancer which allows an identification of these genetic changes which create a varied CNA profile is endometrial cancer. Endometrial cancer has been extensively sequenced by The Cancer Genome Atlas (TCGA) research consortium [8]. The majority of endometrial cancers are curable [9], however a class of Type II endometrial tumors is much more aggressive and less likely to respond to chemotherapy. The TCGA study found aggressive tumors could be identified by unbiased clustering of copy number variation within the tumors. Those tumors with the highest amount of copy number variation indicated the worst prognosis of patients studied in the TCGA cohort [8]. Interestingly, many of these aggressive, copy number variable tumors contained a p53 mutation, but many other p53 mutant tumors had a relatively flat copy number landscape. Since both CNA variable and CNA stable p53 mutant tumors were sequenced, we undertook a study to determine what changes could be contributing to the large copy number variation of aggressive tumors.

Here we investigated the genetic pathways altered by copy number between TP53 mutant uterine endometrial carcinoma tumors with high overall copy number diversity and those tumors with low copy number diversity. The enriched gene sets identified which pathways are involved in the creation of an endometrial tumor’s genetic diversity.

## Results

### Evaluation of low CNA vs. high CNA populations among *TP53* mutant tumors

**Fig 1:**
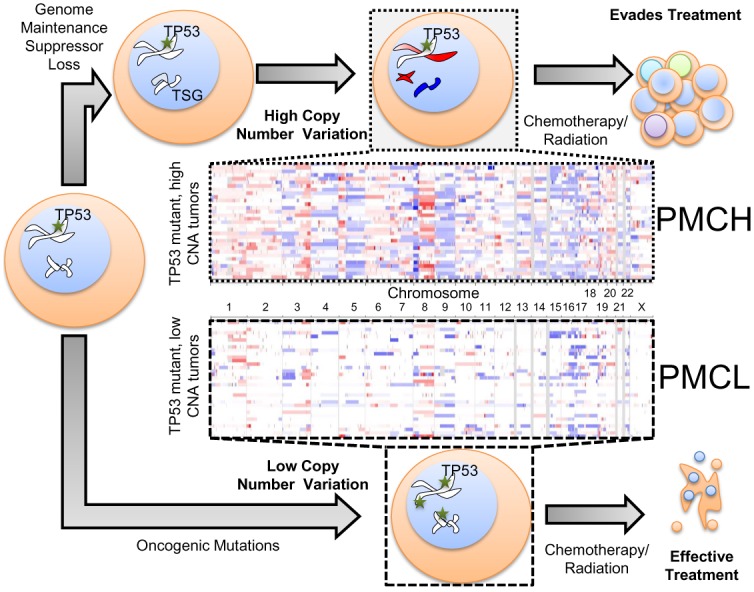
Overview of copy number comparison workflow. *TP53* mutant tumors can derive oncogenic characteristics that contribute to tumor progression via copy number variation (top route) or epigenetic/mutational variation (bottom route). Patient tumors with a *TP53* mutation were segregated into a cohort with either high copy number alterations per tumor (top panel, PMCH) or low copy number alterations (bottom panel, PMCL), initially using median as the cutoff value. Each row represents a single patient’s tumor genetics (N = 34 per group). Blue shading indicates copy number loss, red indications copy number amplification.

For these studies, we used the TCGA Uterine Corpus Endometrial Carcinoma dataset [8], containing 548 patient-tumor pairs. Among the 242 patient tumors with both copy number and mutational data, we focused on the 68 tumors bearing *TP53* mutations (S1 Table). To evaluate specific enrichments among patients with high genomic copy number alterations (CNAs), these tumors were then parsed into those with higher than median CNAs and those with lower than median CNAs (Fig 1). We refer to tumors with p53 mutation and CNAs at high levels as “PMCH” and the tumors with p53 mutation and CNAs at low levels as “PMCL”.

CNA instability has been suggested to be caused by a loss of a tumor suppressor rather than a gain of an oncogene, such as p53 loss or *BRCA1/2* loss. Our analysis focused on genes enriched for losses in PMCH tumors. Ploidy calls derived from the GISTIC2 algorithm [10] for individual gene gain/loss events were used for each gene in each tumor. Our standardized equation (see Materials and Methods) yielded scores for each gene which could be used to rank those genes most often lost in PMCH tumors relative to PMCL tumors. Since monoallelic loss of genes may contribute to tumor formation in a cumulative manner [4], this gene list was then analyzed for statistical enrichments in known molecular pathways. Genes enriched for losses were evaluated via GOrilla [11] for GO term pathway enrichment analysis.

### Key pathways with enhanced depletion among the high CNA *TP53* mutant tumors

**Fig 2:**
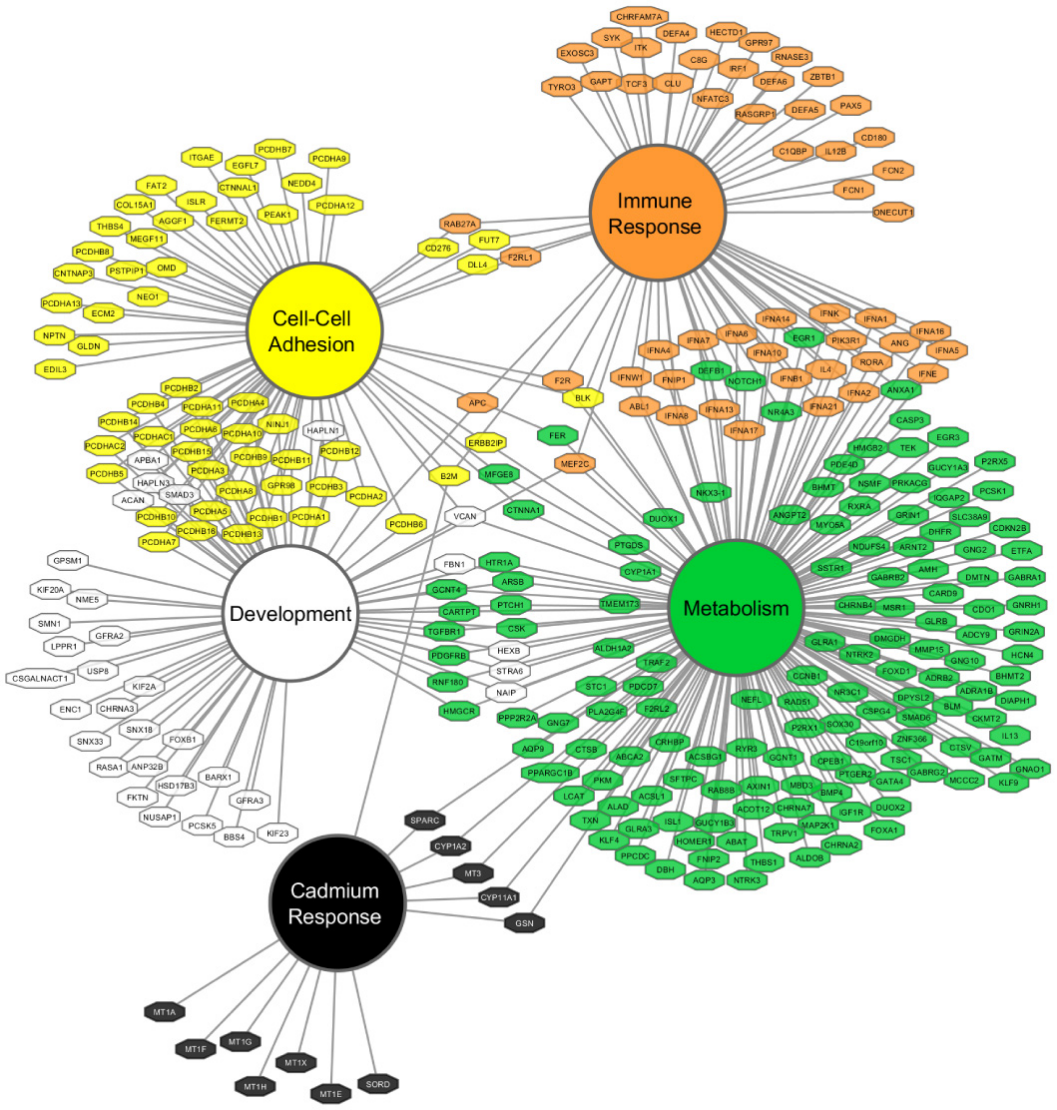
Allelically depleted pathways observed in copy number variable endometrial tumors displayed by GO term. Primary nodes depict GO term pathways enriched for deletions in *TP53* mutant, high copy number variation endometrial cancers. Individual gene nodes depict those genes enriched for deletions in PMCH tumors within annotated GO pathways, with edges connecting genes to pathways enriched for deletions. Note that genes shared between GO terms are linked to each GO term, rather than repeated, for clarity.

A complete list of significant GO terms enriched for losses in PMCH tumors are available in S2 Table. For a detailed description of the statistics used, please refer to materials and methods (section: pathway analysis) and the publication describing the GOrilla statistical tool [11]. The key highlighted pathways are described below with altered genes briefly noted. Many genes are involved in multiple pathways. The interrelation of these pathways is represented in Fig 2.

### Immune System

The somatic alteration patterns analyzed in this study are specific for the tumor and are not alterations in the peripheral blood. Nonetheless, the most significantly enriched pathway for deletions in endometrial tumor cells was that of the immune response. Genes involved in the natural killer cell (*IFNW1*, *IFNA5*, *IFNA6*, *IFNA7*, *IFNA8*, *IFNK*, *IFNA1*, *IL12B*, *IFNA2*, *IFNA4*, *IFNA21*, *IFNA17*, *IFNA13*, *IFNA10*, *IFNA16*, *IFNA14*, *IFNE*, *IFNB1*, *and RAB27A*), B cell (*GAPT*, *CD180*, *PIK3R1*, *FNP1*, *CHRFAM7A*, *ONECUT1*, and *GPR97*), and T cell response (in addition to the interferon genes above, *IL4* and *RORA*) were significantly enriched for deletion/losses among high CNA tumors. Statistical enrichment was q< 3.4x10^-11^.

### Cell-Cell Adhesion

The second most enriched pathway for deletions in high CNA TP53 mutant tumors was that of cell-cell adhesion. Suppressed genes included those in the protocadherin family of integrins (*PCDH* genes), *FAT2* and *DLL4* (which interact with NOTCH signaling), tyrosine kinases *BLK* and *PEAK1*, *ECM2* (a female specific ECM protein), *EGFL7* (an endothelial marker and migration regulator), *FERMT2* (an ECM protein), *B2M* (a MHC I protein often overexpressed in cancer and which can promote metastasis), *NEDD4* (an E3 ubiquitin ligase which negatively regulates PTEN levels), *NEO1* (a FAK interaction protein), *NPTN* (neuroplastin), *CD276* (an immune checkpoint protein), *ITGAE* (an E-cadherin receptor involved in autoimmunity), and *PSTPIP1* (an autoinflammatory response protein). Statistical enrichment was q< 8.4x10^-11^

### Development

The broad class of developmental genes were also prevalent among the high CNA mutant TP53 tumors. Nervous system development (*KIF2A*, *SMN1*, *NAIP*, *ENC1*, *GFRA3*, *CSGALNACT1*, *GFRA2*, *APBA1*, *LPPR1*, *FKTN*, *GPSM1*, *CHRNA3*), mitotic cytokinesis (*SNX18*, *RASA1*, *KIF20A*, *NUSAP1*, *USP8*, *KIF23*, *SNX33*), and general development genes (*HEXB*, *VCAN*, *HAPLN1*, *NME5*, *PCSK5*, *BARX1*, *HSD17B3*, *ANP32B*, *FBN1*, *FOXB1*, *SMAD3*, *BBS4*, *STRA6*, *ACAN*, *HAPLN3*) were enriched for deletions. Statistical enrichment was q< 7.5x10^-6^.

### Metabolism

While less statistically significant, metabolism genes had the greatest overall incidence of losses of any pathway in this analysis. Specifically, metabolic GO terms enriched for deletion included Coenzyme A metabolism (*MCCC2*, *HMGCR*, *and PPCDC*), dsRNA metabolism (*TMEM173*, *CARD9*, *GRIN2A*), oxoacid metabolism (*CKMT2*, *ACTO12*, *ABCA2*, *EFTA*, *ABAT*), serine phosphorylation (*FNIP2*, *TXN*), nitrogen response (many genes), organic cyclic compound response (many genes), S-methylmethionine metabolism (*BHMT* and *BHMT2*), and hormone level regulation (*CARTPT*, *FOXD1*, *GCNT4*, *PCSK1*) including serotonin (*HTR1A*, *RNF180*). Statistical enrichment was q< 2.0x10^-5^.

### Cadmium Response

**Fig 3:**
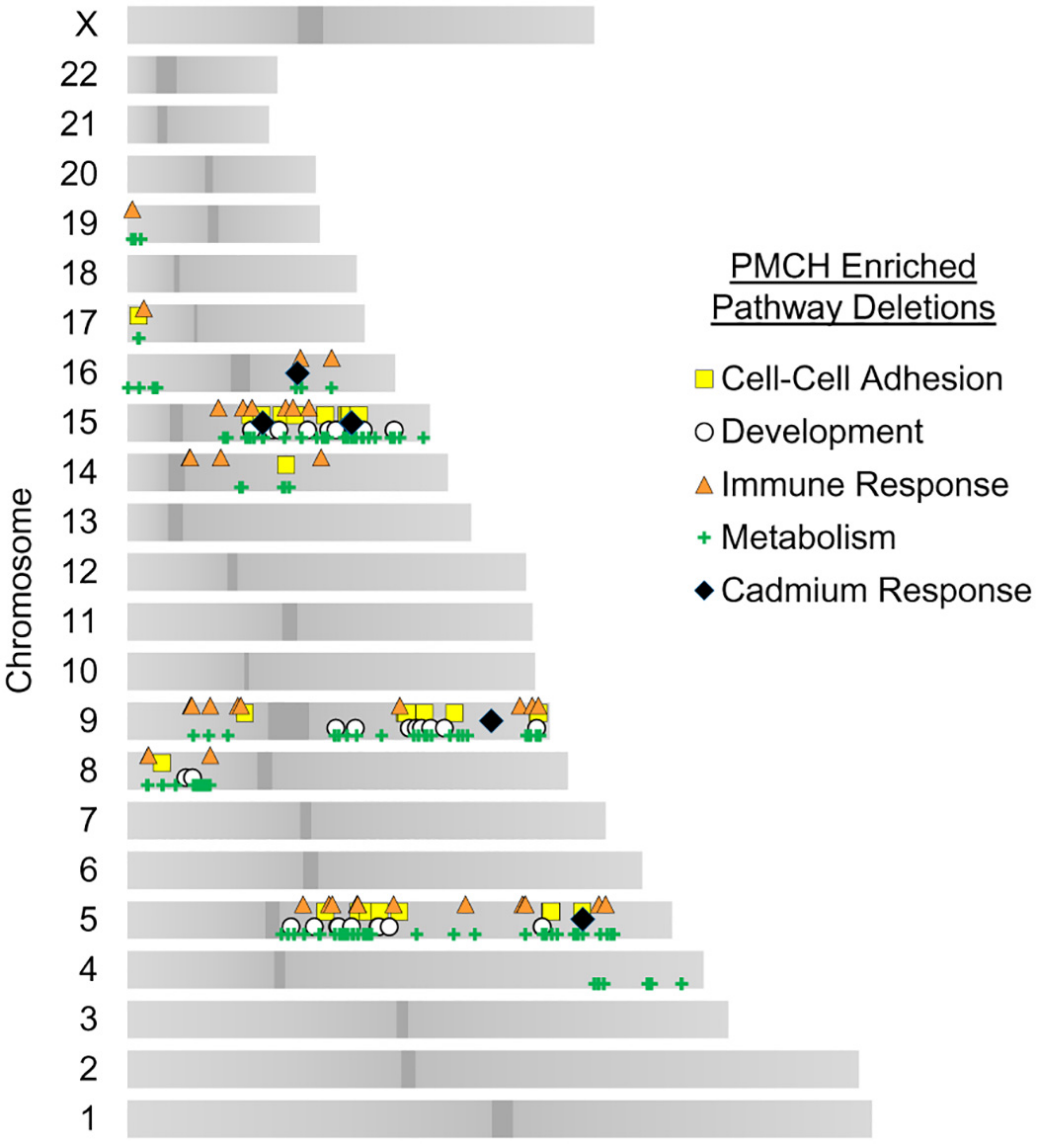
Chromosomal loci of CNA suppressor pathway genes. Locations of gene pathway copy number losses in *TP53* mutant, high copy number variation tumors. Each marker represents an individual gene with an increased incidence of deletion within an annotated GO pathway.

The gene losses in the GO term “cadmium response” (CR) include a swath of genes regulated by heavy metal ions and distributed across the genome; some are in hot spots with other pathway deletions (Fig 3). These regions include 5q (*SPARC* deletion), 9q (*GSN* deletion), 15q (*SORD*, *CYP11A1*, and *CYP1A2* deletions), and a region on 16q containing seven metallothionein genes (*MT3*, *MT1E*, *MT1A*, *MT1F*, *MT1G*, *MT1H*, and *MT1X* deletions). *SPARC*, also known as osteonectin, may be an adhesion suppressing gene and has been linked to cadmium by its specific downregulation as noted following oncogenic exposures of cadmium in bladder cancer [12]. It is expressed, however, following exposure to cadmium in tissues sensitive to the metal [13]. Gelsolin (GSN) is an actin cytoskeleton regulatory protein with severing and nucleating functions that is normally dependent on calcium [14], and translocates to the cytoskeleton following cadmium administration [15] to sever and disrupt cytoskeletal filaments [16, 17]. Sorbitol dehydrogenase (SORD) converts sorbitol to fructose. Its levels are upregulated by cadmium exposure [18], possibly due to the fact that the enzymatic activity is decreased by cadmium exposure [19]. The cytochromes Cyp11a1 and Cyp1a2 are involved in detoxification of numerous xenobiotics. The metallothionein (MT) proteins have a high affinity for cadmium and sequester the majority of cadmium in the body [20], including in endometrial cells [21]. The loss of 16q in CNA high uterine endometrial carcinomas induces a loss of a cluster of seven of these metallothionein genes, suggesting a substantial suppression in the cells’ ability to cope with heavy metal exposure. Statistical enrichment was q< 3.1x10^-3^.

### Genes with CNA display both focal and widespread chromosomal distributions

Whole chromosome and chromosome arm losses are known, widely prevalent mechanisms for CNAs in cancer. These gross losses and gains are thought to encompass multiple tumor suppressors and oncogenes to cumulatively affect cell cycle dysregulation and other tumor formation characteristics [4]. *In vivo* studies have validated that knockdown of genes within gene clusters independently contribute to oncogenesis[22]. Even the most commonly inactivated tumor suppressor, *TP53*, may be commonly deleted due to neighboring tumor suppressors *EIF5A* and *ALOX15B[23]*. Since some biological pathways utilize many genes within a short region of the genome in arrayed gene sets, it is possible some of our GO term enrichments were found due to local clustering. To address this possibility, we mapped the genes enriched for loss in PMCH tumors to their chromosomal locations and graphed their distribution (Fig 3). We found that the pathways most enriched for gene loss spanned regions across multiple chromosomes, suggesting that the metabolic, developmental, cell-cell adhesion, and immune response pathways involve losses of more than just one chromosome or chromosome arm.

However, the CR pathway enriched by GO term analysis contained 7 out of 12 total genes in a focal array on cytoband 16q13. All these genes encode metallothionein proteins involved in the sequestering of cadmium and other xenobiotic heavy metals within the cell. At a first glance, we formed the hypothesis that because of this focal arrangement of genes, the CR pathway may be a false positive from the GO term enrichment analysis. However, there has been much debate in epidemiology regarding how exposure to cadmium influences endometrial cancer incidence. A 2008 study provocatively suggested cadmium contaminated vegetables and cereals increases endometrial cancer risk by 39% [24]. Follow-up by two prospective cohort studies suggested a positive trend of cadmium exposure as a risk factor for endometrial cancer in women with a BMI<25 in one [25], while the other found no association [26] possibly due to apparent difficulty in accurately estimating biological exposure to environmental cadmium. A meta-analysis of these studies and others concluded cadmium exposure is a risk factor in subsets of the population [27]. From a molecular biology standpoint, cadmium has been described as a mutagen in part due to its creation of reactive oxygen species [28], mismatch repair inhibition [29], and from its ability to cause double-stranded DNA breaks [30, 31]. Taking these previous findings into consideration, we decided not to rule out the CR pathway as a possible contributor to PMCH tumor formation and continued to incorporate these genes into subsequent analyses.

### Early vs. Late Loss Events in Tumor Development

The relative prevalence of genetic alterations within a tumor sample can help indicate if an alteration occurred ‘early’ or ‘later’ during the development and adaptation of a tumor. Mutations, in particular, are widely used to determine “trunks” of the oncogenic development tree of a tumor; those mutations which are prevalent in near 100% of the tumor sample are more likely to have occurred early, and those of lower prevalence likely occur later in tumor development. In technical terms, mutation prevalence is calculated as a fraction of sequence reads; the smaller the fraction the fewer tumor cells are presumed to contain the mutation. Similarly, prevalence of copy number changes may be inferred from the log_2_ ratio of signal resulting from SNP array data targeting a gene: the higher the signal the fewer cells have acquired or retained the gene deletion. However, this can be confounded by the fact that it is difficult to determine if a loss is homozygous in half of the sample or heterozygous for the entirety of the sample. However, if alterations are mostly heterozygous, as determined by the GISTIC2 algorithm [10], then approximations of earlier tumorigenic changes can be calculated.

**Fig 4:**
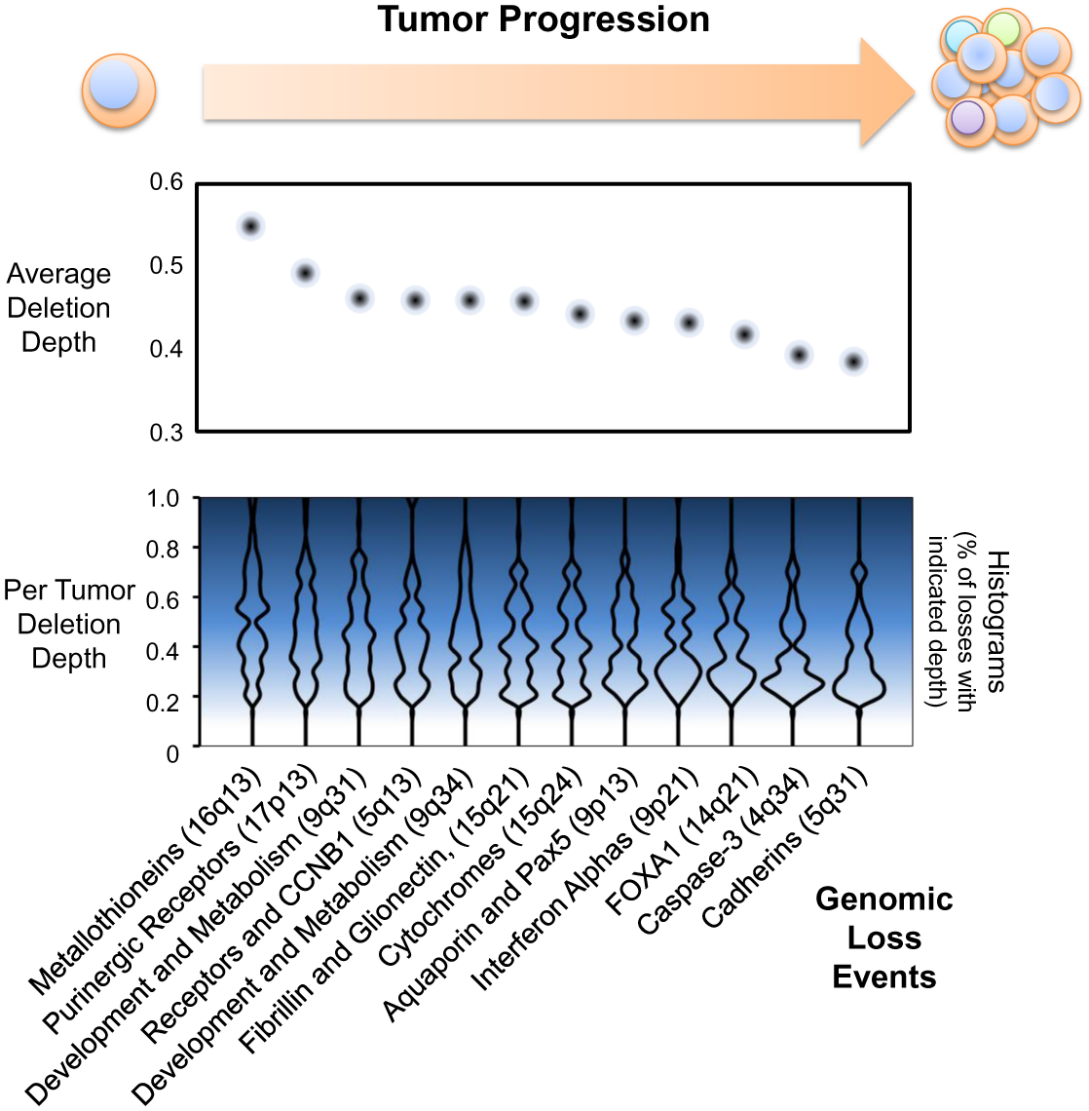
Temporal loss of chromosome regions in PMCH tumor progression. Histograms of copy number alteration magnitudes are generated from tumors with a -0.2 or greater decrease in genomic signal for the indicated genomic regions; results are mirrored for emphasis in this graphical representation. Dots indicate median magnitude, or “deletion depth”, of copy number loss across all tumors with the indicated loss event. Based upon depth of loss (see methods) the cadmium response metallothionein genes appear to be lost prior to other copy number events (p<1x10^-11^ compared to all other regions, p<0.03 compared to cytoband 17p13).

This assumption proved to be reasonable; all genes in Figs 2 and 3 enriched for losses had a frequency of homozygous deletions in <1% of tumors, with the exception of deletions on chromosome 8p. Chromosome 8p is already known to contain many tumor suppressors, such as *DLC1* and *FGL1* [22]. To find novel tumor suppressors, we therefore constructed (see Materials and Methods) a ranked intratumor temporal loss series of all genes enriched for losses by GO pathway analysis (S1 Fig). A consolidated view of these genes is presented in Fig 4.

The deep loss of region 16q13 encompasses the seven metallothionein genes as well as other potential tumor suppressors, such as mIR-138-2 [32], indicating that loss of this region appears to be an early event in PMCH tumors and likely contributes to the formation of further copy number variation. The next most present loss was 17p13, which incorporates just five genes enriched by GO pathway analysis: *P2RX5* and *P2RX1*, encoding ligand gated Na^+^/Ca^2+^ ion channels regulated by purinergic signaling, *TRPV1*, a non-selective ion channel, *ITGAE*, an integrin involved in E-cadherin ligation, and *C1QBP*, a poly-phenotypic protein that is commonly associated with tumor progression [33] but also involved in increased glycolysis and the Warburg effect [34]. Interestingly, the P2X receptor activity is either augmented or blocked by bound cadmium, depending on the receptor, suggesting a possible interaction with the cadmium response [35, 36].

The majority of regions were lost at similar rates across cells in a tumor (Fig 4), suggesting once CNAs variation has been enabled, a number of pathways then enable tumor cells to undergo positive Darwinian selection. These regions were involved in all enriched aspects of PMCH tumors, including metabolism, development, and cell-cell interactions. Somewhat unexpectedly, some of the latest events in tumor progression incorporated caspase-3, an executioner caspase downstream of apoptosis initiation. Cadherin deletions on 5q31 (including 31 members of the protocadherin family) were the least prevalent losses in the tumor cell population, consistent with the idea that metastatic ability increases later in tumor development. Since the tumors analyzed were primary tumors extracted prior to therapy, one might have tested the prediction that cadherin deletions are much more enriched in metastatic tumor samples. However, these data were not available.

### Effect of cadmium response gene loss is specific to CNAs, not mutations

Since metallothionein genes were the earliest loss events in our analysis, we characterized the capacity of CR gene loss to promote ongoing tumor mutagenesis. To address whether or not CR gene loss acts to alter mutation rates in addition to copy number alterations, we utilized the COSMIC database [37] to compare mutation types and rates between cadmium gene suppressed patients and those patients without CR gene loss in the *TP53* mutant cohort.

**Fig 5:**
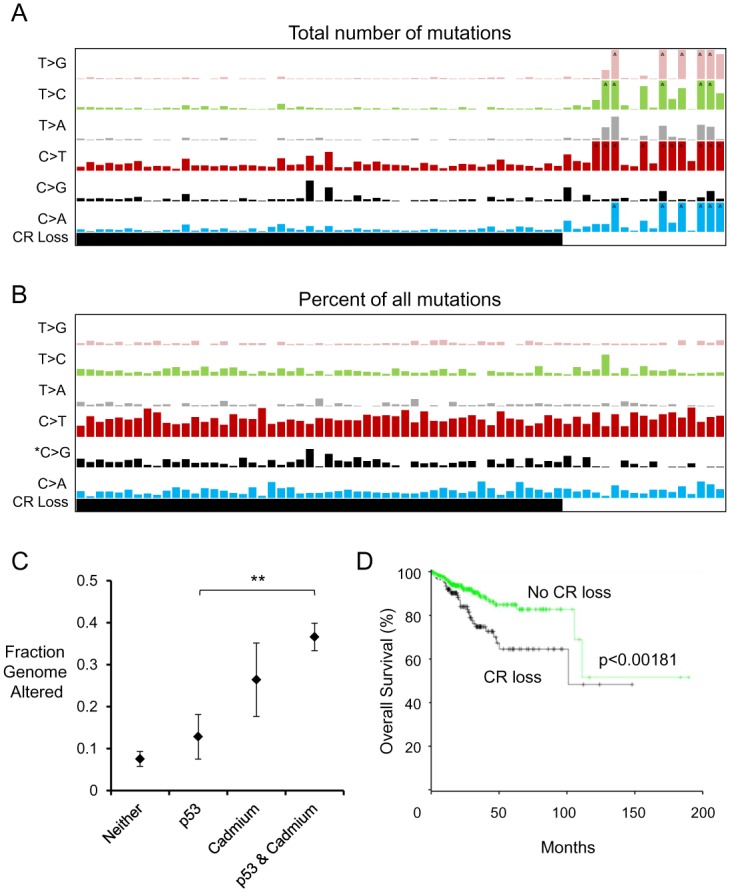
Genetic alteration diversity patterns in CNA high and low tumors. (A) Number of exonic mutations in *TP53* mutant tumors with or without cadmium response (CR) gene loss. Highest bars in this representation correspond to 100 mutations (^ indicates more than maximum on graph) (B) Percentage of exonic mutations as in (A), normalized for number of total mutations. Highest bars in this representation correspond to 75% of all mutations (C) Comparison of tumor copy number changes in patients with *TP53* mutation, cadmium response gene loss, or both. **p<0.01 (D) Loss of cadmium response genes indicates worse prognosis in endometrial cancer. Patient cohort shown is independent of *TP53* mutation.

We found that early loss of CR was associated with decreased mutagenesis relative to tumors that maintained CR genes. In fact, the tumors which did not exhibit losses of CR genes had far higher mutation rates across all mutation types (Fig 5A), consistent with *POLE* and microsatellite mutations driving tumorigenesis in many endometrial cancer patients[8]. The spectrum of mutations did not change in these cohorts, although there was a slight statistically significant enhancement of C>G mutations in tumors with CR gene loss (Fig 5B).

In contrast to the mutation rates observed, we confirmed the strong correlation between cadmium response gene loss and fraction of the genome which was altered in UCEC tumors. About 36% of the tumor genome had copy number changes in the CR gene loss *TP53* mutant cohort, while only 13% was altered in *TP53* mutant tumors without CR losses (p< 0.01 by Wilcoxon rank-sum test) (Fig 5C). Taken together, these results suggest loss of genomic regions containing CR genes most likely results in copy number instability rather than mutational instability. These changes are clinically significant; patients with losses in CR genes have nearly a doubled hazard ratio resulting in negative prognosis (Fig 5D). Such data fit the histopathological understanding that the serous subtype is generally more lethal. Accordingly: 71% of the PMCH cohort were annotated within the serous histological subtype, whereas only 47% of the PMCL cohort were serous, suggesting copy number variation contributes to the serous phenotype of p53 mutant endometrial tumors.

## Discussion

Here we investigated the genomic cause for copy number variation in the most aggressive subtype of endometrial cancer. There was a divergence in *TP53* mutant tumors: those which evolved primarily by acquiring mutations (PMCL cohort) and those which evolved in part by acquiring copy number changes (PMCH cohort). Our gene pathway analysis of regions enriched for deletions in PMCH tumors revealed that immunological, metabolic, developmental, adhesion and matrix signaling, and cadmium response pathways are enriched for deletions. The CR pathway loss, in particular the loss of metallothionein genes, occurred earlier in tumor evolution than any other regional loss. Tumors with genetically suppressed CR largely did not alter their mutational spectrum or increase mutational load, but rather increased the amount of copy number changes by 186% (Fig 5).

While we were expecting to find more canonical DNA maintenance pathways enriched in our GO term datasets, we were surprised to find CR genes as the earliest hit and a prognostic indicator of overall survival. Cadmium is a well-known carcinogen. The influence of cadmium on endometrial cancer incidence has been somewhat controversial; epidemiology studies have found correlations of increased incidence of endometrial cancer with high dietary exposure to cadmium in some [24, 25], but not other [26] prospective studies. A common suggestion in the cases which did not find a significant association (there was always a trend) is an inability to accurately measure cadmium exposure. Our results suggest that the patients with high copy number variation and poor prognosis have experienced an early evolutionary event in their tumor cells which have suppressed endometrial cells’ ability to sequester cadmium within metallothioneins. In addition, this provides a biological mechanism for how endometrial cancer and cadmium exposure may interact. Cadmium can induce aneuploidy in multiple model systems [38], including mouse oocytes [39]. It may do so by increasing the amount of reactive oxygen species, which in endometrial cancer would be further exacerbated by cytochrome deletions. Cells in the uterus with a loss in the CR pathway would selectively uptake cadmium without any sequestration from molecular machinery, allowing this carcinogen to wreak further havoc and initiate oncogenesis. While this link of metallothionein proteins to cadmium appears important, it should be noted that metallothioneins sequester a number of heavy metal ions which may impact endometrial biology [40, 41]. Metallothioneins also act to buffer zinc [42], impacting both enzymatic and transcriptional activities within the cell. [43]. Metallothioneins may also regulate oncogenesis in other cancer types such as serous ovarian cancer and breast cancer, which are deleted for metallothioneins in 68% and 59% of cases, respectively [44].

Since the analysis presented here is observational, it remains to be directly shown that polygenic pathway losses in UCEC are causal for a phenotype. There are, however, many documented cases where loss of a single allele of a single gene, without a mutation in the other allele, produces an increase in tumorigenesis. *PTEN* loss is present in many cancer types, and mouse studies have a demonstrable increase in spontaneous tumor formation [45]. *BECN1*, an autophagy initiation gene also involved in ploidy homeostasis [46], increases tumor formation when monoallelically deleted [47, 48]. The RNAi knockdown of multiple chromosome 8p tumor suppressor homologues in mice can incur oncogenesis [22]. From our study, we would predict loss of metallothionein genes to sensitize mice or human tissue to oncogenesis from cadmium exposure.

While loss of CR appeared earliest in the tumor, the remainder of the enriched pathways are in fractions of cells and may contribute to tumor presentation and micrometastasis. Interferons are normally expressed in cells infected by viruses or bacteria to recruit the immune system via MHC presentation to cytotoxic T cells [49]. A simultaneous deletion of 17 of these interferons occurs in PMCH tumors and may assist immune evasion. Metabolic and developmental alterations may allow for further growth of the tumor in hypoxic or physically constrained environments.

One advantage of monoallelic changes for tumor cells is an ability to retain oncogenes for later stages of tumor development, including metastasis. For example, loss of chromosome 9q34 removes tumor suppresser genes *TSC1* (an mTOR pathway inhibitor [50]), *NOTCH1* (a development and stemness regulator [51]), and *GRIN1* (a calcium regulating tumor suppressor [52]). But deletion of 9q34 also removes *ABL1*, a potent oncogene when upregulated [53], as well as *RXRA*, an oncogene which can be activated by SUMOylation [54]. However, since both alleles are not deleted, these oncogenes are not completely removed and can be epigenetically or post-translationally up-regulated if necessary. This is analogous to the gene *BECN1* and the regulation of autophagy. Beclin, the product of *BECN1*, promotes the formation of autophagosomes and recycling of cellular constituents. *BECN1* is a haploinsufficient tumor suppressor [47, 48], yet once a tumor forms and expands, tumors require autophagy for essential metabolites, especially in hypoxic or otherwise nutrient-poor tumors [55, 56]. Examples like 9q34 show how a relatively short region of a single chromosome can contain many tumor suppressors and oncogenes, and each tumor regulates their proportional dosage to favor growth.

Challenges surrounding genomic cancer experiments are progressing from the initial simple difficulty of obtaining genetic information to those difficulties associated with interpretation and applying genomic information [57]. This study supports the idea that pathway analysis from the entire genome, rather than individual gene or chromosome arm analysis, contributes to our understanding of the pathogenesis of cancers. Future studies will be needed to determine causality regarding the potential tumor suppressor genes and pathways found here to be enriched for losses.

## Materials and Methods

### Patient cohort

The NCI Cancer Genome Atlas cohort was utilized for this study, due to its consistent data structure and large sample size [8]. Tumors incorporated into their study post-publication were included in this analysis, for a total of 548 patients, 242 of whom had both mutation and CNA data, and 68 of these with a *TP53* mutation. These patients were defined as having a diagnosis of endometrioid adenocarcinoma or endometrioid serous carcinomas. All patients must have required surgery which extracted the tumor, and no patients at the time of surgery had detectable metastatic lesions. No prior chemotherapy or radiotherapy was allowed for incorporation into this dataset. Somatic alteration determination was made possible by paired sequencing of normal tissue from the patient, either from blood or from tissue >2cm from the tumor. We defined tumors with p53 mutation and CNAs at higher than median levels as “PMCH” and the tumors with p53 mutation and CNAs at lower than median levels as “PMCL”. Median age of patients with PMCH and PMCL tumors were both 68 years.

### Mutation and copy number alteration (CNA) determination

All data were extracted and analyzed from The Cancer Genome Atlas (TCGA) consortium’s sequencing of Uterine Corpus Endometrial Carcinoma (UCEC) patient-tumor pairs, as maintained in the UCSC Cancer Genome Browser. The provisional dataset of 548 tumors, 242 of which had both CNA and mutation data, were downloaded and analyzed for this study. *TP53* mutant tumors were identified as those annotated with a p53 mutation in cBioPortal [44], which were 68 tumors. The total fraction of the genome altered (Fig 5C) were downloaded from the curated values within the clinical characteristics in cBioPortal, as were survival data. Survival curves compared patients with 16q13, 9q34, or 15q24 losses (cadmium response regions), or those without these losses, independent of p53 status. TCGA copy number signals were generated on Affymetrix SNP 6.0 arrays and run through the Broad Institute pipeline [58] to determine CNVs, and we used these data as curated by the UCSC Cancer Genome Browser [59]. GISTIC2 copy number calls were used to assess allelic losses [10]. SNP data were input into the Integrated Genomics Viewer [60] to generate the loss/amplification panels of PMCH and PMCL tumors in Fig 1.

### Assessment of enriched losses in PMCH tumors

We used the following equation to determine loss enrichment scores for a given gene *α*:

$$\left(-PMCH\overset{j}{\underset{i}{\sum }}\alpha GISTIC\right)-\left(-PMCL\overset{l}{\underset{k}{\sum }}\alpha GISTIC\right)=\alpha LOSS$$

where gene scores are summed from all patient tumors within a genotype group PMCH or PMCL. α_GISTIC_ scores are gene specific GISTIC2 [10] scores which range from: -2 (biallelic or complete loss), -1 (allelic loss), 0 (no change), 1 (allelic amplification), 2 (biallelic or more amplification). This strategy incorporated an equal weight against gains of tumor suppressors, since genes acting as dosage dependent tumor suppressors in the development of PMCH tumors should have a reduced frequency of gain events in conjunction with an increased frequency of loss events. Therefore, a gene with a high α_LOSS_ score is a gene deleted in many more PMCH tumors than PMCL tumors, and a gene with a very low α_LOSS_ score is a gene deleted more often in PMCL tumors or a gene amplified in PMCH tumors.

### Pathway analysis

Ranked α_LOSS_ scores were then input directly in GOrilla [11] and output GO terms are summarized in S1 Table. This strategy results in GO term hits for pathways which contain genes commonly deleted in PMCH tumors relative to PMCL tumors. Since we utilized a ranked gene list based on genes lost in decreasing enrichment in PMCH tumors, the hypergeometric score output from the algorithm was used. Only those GO terms with a false discovery rate adjusted q value of <0.05 were placed in S1 Table. GOrilla improves on some competitor GO term analyses from its ability to incorporate a user-based background gene set, rather than an arbitrary background list of annotated genes, in its calculations for enrichment. Our background set included all genes with copy number data throughout the TCGA UCEC study. GO terms were manually placed into the five overarching groups using annotated hierarchies from the Gene Ontology consortium [61]. For any gene within more than one enriched GO term, a literature search was performed to determine which pathway color is most relevant, although multiple-pathway annotations are retained in the network depiction in Fig 2 as multiple edges. Cytoscape was used to produce the graphical network display in Fig 2.

### Genomic location mapping

Whole genome gene IDs were input into bioDBnet [62] and converted into Ensembl annotated chromosome locations. Centromere locations were downloaded from the UCSC human genome browser (hg19). Genes output from GOrilla analysis were then sliced from this dataset and graphed by Microsoft Excel.

### Temporal assessment of chromosome region losses

Raw reference normalized SNP signal was downloaded from the UCSC Cancer Genome browser, sliced for those tumors and genes under evaluation, and compared to GISTIC2 copy number calls. All GISTIC2 calls of -1 in any tumor were included in the analysis. SNP values were used to determine timing of region loss using the following assumptions: only monoallelic, not biallelic, losses occurred (chromosome 8p did not fit this assumption and was excluded), the stronger the frequency of the variant the earlier the loss (as it is present in more cells within the sample), and variants of a log2 ratio of <0.2 were potential noise events and excluded from analysis. All genes resulting from GO term enrichment analysis, as depicted in Figs 2 and 3, were included in our analysis (S1 Fig). Histograms of each chromosome region in Fig 4 incorporate minimum SNP values of the queried region across all UCEC tumors with CNA data, and are mirrored for presentation purposes. Median SNP values of all GISTIC2 “-1” value regions are shown for comparison in Fig 4 (top panel). Overall, this approach mirrors the method used for mutational evolution of tumors by using the Cancer Cell Fraction within a sample [1].

### Base-pair scale mutational analysis

For determination of transitions and transversions detected in whole-genome sequencing, we downloaded the COSMIC database [37] of cancer mutations and sliced the UCEC dataset for analysis. Mutation scores were summed for each individual tumor and mapped to *TP53* mutation and/or Cadmium Response Loss cohorts, as described above. Tumors with a loss in any of the 12 genes enriched of loss in the Cadmium Response pathway were counted in the “CR” bar of Fig 5.

## Supporting Information

S1 Fig Loss depth of all GO enriched gene deletions by pathway. Stacked histograms of copy number alteration magnitudes are generated from tumors with a -0.2 or greater decrease in log_2_ genomic signal for the indicated genes. Each pathway panel is sorted left to right from highest to lowest median deletion depth. *Indicates genes on chromosome 8p, which may be confounded by an artificially high signal due to populations of cells with homozygous deletions. Some clusters of highly related and physically adjacent genes are collapsed into a single stacked column for clarity: MT (*MT1X*, *MT3*, *MT1A*, *MT1F*, *MT1G*, *MT1H*, *MT1E*), PCDHB (*PCDHB1-8*, *PCDHB16*, *PCDHB10-15*), PCDHA (*PCDHA5-9*, *PCDHA12-13*, *PCDHAC1-2*), IFNA (*IFNA1-2*, *IFNA5-6*, *IFNA8*, *IFNA13-14*).
(PDF)

S1 Table Patient and tumor characteristics of *TP53* mutant UCEC tumors. The table lists the sample identifier, p53 mutation status, as well as the tumor type classification, number of annotated mutations and the other diagnostic criteria for the clinical data used in the study.
(XLSX)

S2 Table Complete list of GO terms found to be enriched in PMCH tumors. The table lists the GO term, with their brief description, followed by the both q and p vales for significance of the observed losses and a listing of the genes included. Note that some genes are listed to impact more than one pathway, and appear more than once in the table.
(XLSX)
