# Supplementary figures and images for: Whole Genome Pathway Analysis Identifies an Association of Cadmium Response Gene Loss with Copy Number Variation in Mutant p53 Bearing Uterine Endometrial Carcinomas
Source: PLoS One. 2016 Jul 8;11(7):e0159114. doi: 10.1371/journal.pone.0159114 (PMC4938382; doi:10.1371/journal.pone.0159114)

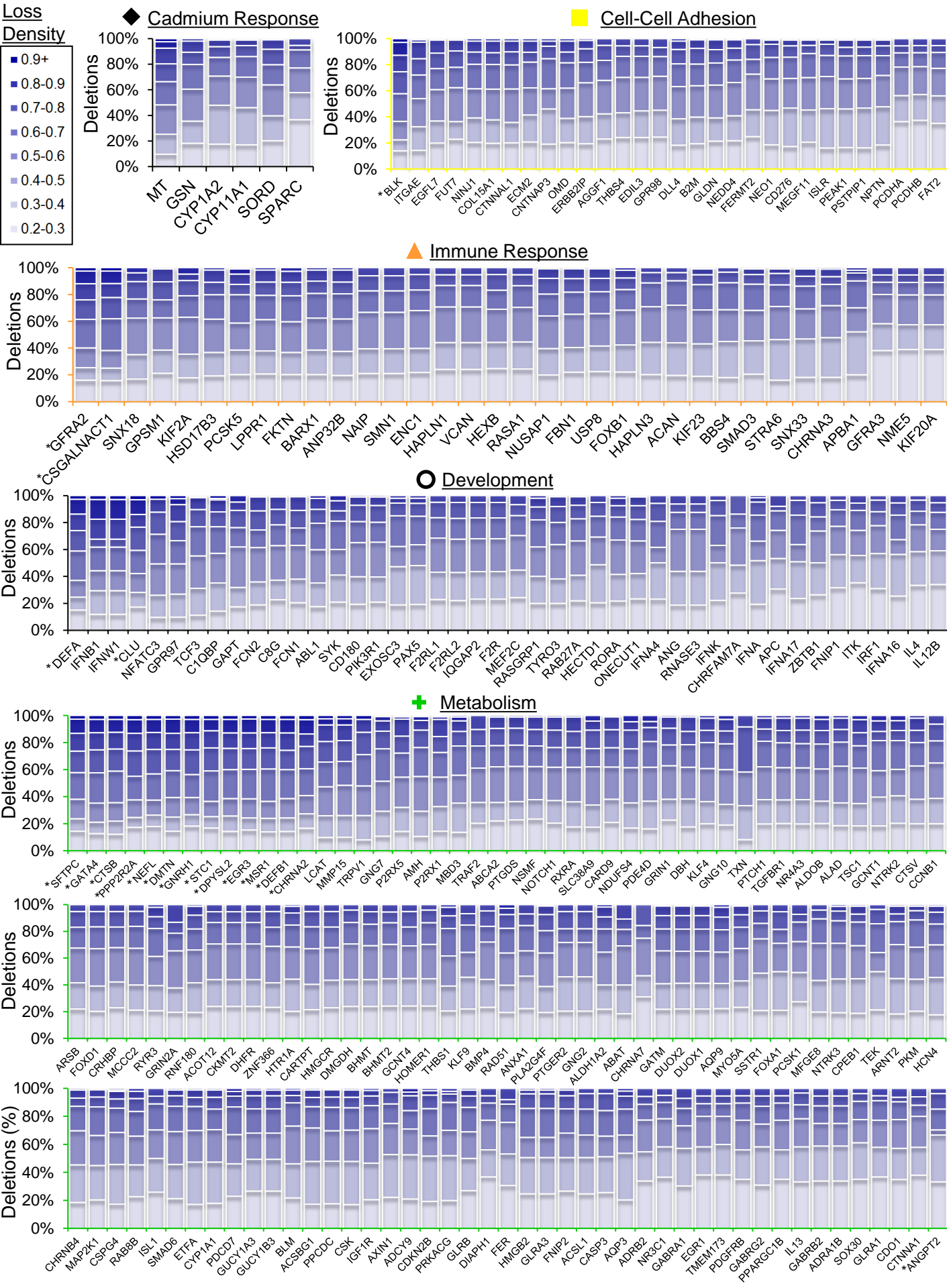

Supplement: S1 Fig — Stacked histograms of copy number alteration magnitudes are generated from tumors with a -0.2 or greater decrease in log2 genomic signal for the indicated genes. Each pathway panel is sorted left to right from highest to lowest median deletion depth. *Indicates genes on chromosome 8p, which may be confounded by an artificially high signal due to populations of cells with homozygous deletions. Some clusters of highly related and physically adjacent genes are collapsed into a single stacked column for clarity: MT (MT1X, MT3, MT1A, MT1F, MT1G, MT1H, MT1E), PCDHB (PCDHB1-8, PCDHB16, PCDHB10-15), PCDHA (PCDHA5-9, PCDHA12-13, PCDHAC1-2), IFNA (IFNA1-2, IFNA5-6, IFNA8, IFNA13-14). (PDF) [file pone.0159114.s001.pdf]
